# Effect of Sheng-Jiang Powder on Gut Microbiota in High-Fat Diet-Induced NAFLD

**DOI:** 10.1155/2020/6697638

**Published:** 2020-11-26

**Authors:** Juan Li, Qian Hu, Dai Xiao-yu, Lv Zhu, Yi-fan Miao, Hong-xin Kang, Xian-lin Zhao, Jia-qi Yao, Dan Long, Wen-fu Tang, Mei-hua Wan

**Affiliations:** ^1^Department of Integrated Traditional Chinese and Western Medicine, West China Hospital, Sichuan University, Chengdu 610041, Sichuan Province, China; ^2^Key Laboratory of Transplant Engineering and Immunology, Sichuan University, Chengdu 610041, Sichuan Province, China

## Abstract

**Background and Aims:**

Nonalcoholic fatty liver disease (NAFLD) is an alarming global health problem that is predicted to be the major cause of cirrhosis, hepatocellular carcinoma, and liver transplantation by next decade. Gut microbiota have been revealed playing an important role in the pathogenesis of NAFLD. Sheng-Jiang Powder (SJP), an empirical Chinese medicine formula to treat NAFLD, showed great hepatoprotective properties, but the impact on gut microbiota has never been identified. Therefore, we performed this study to investigate the effect of SJP on gut microbiota in NAFLD mice.

**Methods:**

NAFLD was induced by 12 weeks' high-fat diet (HFD) feeding. Mice were treated with SJP/normal saline daily for 6 weeks. Blood samples were obtained for serum biochemical indices and inflammatory cytokines measurement. Liver tissues were obtained for pathological evaluation and oil red O staining. The expression of lipid metabolism-related genes was quantified by RT-PCR and Western blotting. Changes in gut microbiota composition were analyzed by the 16s rDNA sequencing technique.

**Results:**

HFD feeding induced significant increase in bodyweight and serum levels of TG, TC, ALT, and AST. The pathological examination revealed obvious hepatic steatosis in HFD feeding mice. Coadministration of SJP effectively protected against bodyweight increase and lipid accumulation in blood and liver. Increased expression of PPAR*γ* mRNA was observed in HFD feeding mice, but a steady elevation of PPAR*γ* protein level was only found in SJP-treated mice. Meanwhile, the expression of FASN was much higher in HFD feeding mice. Microbiome analysis revealed obvious changes in gut microbiota composition among diverse groups. SJP treatment modulated the relative abundance of short-chain fatty acids (SCFAs) producing bacteria, including norank-f-*Erysipelotrichaceae* and *Roseburia*.

**Conclusions:**

SJP is efficient in attenuating HFD-induced NAFLD, and it might be partly attributed to the regulation of gut microbiota.

## 1. Introduction

Nonalcoholic fatty liver disease (NAFLD) is an alarming global health problem that is predicted to be the major cause of cirrhosis, hepatocellular carcinoma, and liver transplantation by 2030 [1, 2]. As the leading chronic liver disease, NAFLD is directly associated with the rising epidemic of obesity and causes huge social-economic burden impacting almost one quarter of the world's population [3, 4]. Characterized by excessive fat deposition in hepatocytes, NAFLD comprises a wide spectrum of liver conditions varying in severity of injury, ranging from simple steatosis to steatohepatitis, fibrosis, and finally cirrhosis [5]. However, the pathogenesis of NAFLD has not been fully addressed yet. A “two-hit” theory has been widely accepted for several years, and it suggests that a second hit from factors like inflammatory cytokines, lipid peroxidation, mitochondrial dysfunction, and especially oxidative stress leads to steatohepatitis in the setting of “first hit” involving simple hepatocyte steatosis [6, 7]. Actually, steatohepatitis is not always progressed from simple steatosis, and there are a variety of molecular pathways which bring about steatohepatitis [8, 9]. Therefore, this theory is currently considered outdated and gradually replaced by a “multiple hit” theory [10] which reflects the interactions between multiple injuries, including insulin resistance, inflammatory cytokines, hereditary and epigenetic factors, oxidative stress, environmental and dietary factors, as well as gut microbiota [11]. Particularly, a growing body of evidence has revealed the important role of altered gut microbiota in the development of NAFLD.

There are trillions of the microbes colonized in the gastrointestinal tract, and the amounts and composition of gut microbiota are stable under normal conditions. Host environmental changes, including diet, alcohol intake, antibiotic abuse, and hereditary factors, may lead to dysbiosis of gut microbiota that intimately related to hepatic lipid accumulation and development of all stages of NAFLD [12]. Germ-free animals have been employed to define the impact of gut microbiota on the host physiological functions for decades. Accumulated evidences have shown that the absence or presence of these bacteria influences hepatic gene expression, calorie usage, lipid metabolism, insulin resistance, and cytokines production, which highlights the probable role of gut microbiota in NAFLD [13, 14]. Le Roy et al. [15] were the first to confirm that the gut microbiota are determinate to the development of NAFLD. They selected two donor mice showing different responses to high-fat diet and observed the consequent pathological response after their microbiota were transplanted into germ-free mice. Their results suggested a different gut microbiota composition in HFD-sensitive mice that finally developed NAFLD compared to HFD-resistant mice without any NAFLD features. And the propensity to develop NAFLD was transmissible as they occurred only in those who transplanted with the microbiota of NAFLD mice. However, the relationship of gut microbiota and NAFLD might be bidirectional as substantial evidences have disclosed obvious alterations in the composition of gut microbiota in NAFLD patients and mice compared to that of healthy individuals [16, 17]. All these discoveries highlighted the critical role of gut microbiota in the development of NAFLD and pointed out the potential of a gut microbiota-targeted therapeutic strategy.

Today, the fundamental treatment for NAFLD is lifestyle intervention as there is no licensed pharmacotherapy specific for NAFLD [18]. But actually, most people find it hard to adhere to lifestyle change whether it is physical activity implement or dietary restriction. So researchers are on the way of finding new agents to treat NAFLD. Numerous drugs have been tested for NAFLD treatment so far. These drugs are classified into several categories according to their pharmacological effect, including antioxidants, antidiabetic drugs, lipid lowering drugs, and prebiotics [19]. The above classes of drugs may have shown benefit for NAFLD controlling, but we are clearly aware of that it is difficult to find a pharmacological agent that targets wide ranging of the previous described complex physiopathology of NAFLD. Accordingly, the traditional Chinese herbal medicine shows great potential in treating NAFLD and preventing disease progression due to the multicomponent, multitarget, and multipathway properties. Emerging evidences have proved their role in regulating lipid metabolism, reducing oxidative stress, ameliorating insulin resistance, and modulating gut microbiota [20–22]. Given the pathogenesis of NAFLD, the theory of traditional Chinese medicine attributes NAFLD to a category of “liver turbidity” and the essential pathogenesis of NAFLD is disordered motion of qi, which further causes qi stagnation, phlegm retention, and blood stasis, and then induces the development of NAFLD. As a classic representative traditional Chinese medicine formula, Sheng-Jiang Powder (SJP) showed satisfying clinical effect in treating NAFLD with confirmed wide ranging of pharmacological effects, such as anti-inflammation, lowering body weight, mitigating insulin resistance, and regulating immune response [23]. Also, our previous study has demonstrated that SJP could effectively prevent HFD-induced weight gain and hepatic lipid accumulation and subsequent liver injury via regulating lipid metabolism-related pathways [24]. However, we have not had any knowledge about the impact of SJP on gut microbiota so far. Considering the critical role of gut microbiota on the development of NAFLD, the present study was designed to explore the effect of SJP on gut microbiota by using a mice model of NAFLD.

## 2. Materials and Methods

### 2.1. Design

A prospective, randomized controlled trial was conducted.

### 2.2. Settings

The study was conducted at Key Laboratory of Transplant Engineering and Immunology, West China School of Medicine, Sichuan University.

### 2.3. Ethics Statement

The protocol was approved by the Ethics Committee for Animal Experiments of Sichuan University. All mice were handled according to the University Guidelines and the Animal Care Committee Guidelines of West China Hospital. All efforts were made to minimize suffering of mice.

### 2.4. Preparation of Sheng-Jiang Powder

The preparation of SJP was described in our previous studies [25]. In brief, the spray-dried powder purchased from Chengdu New Green Herbal Pharmaceutical Co., Ltd. (Chengdu, China) was mixed together according to the original compatibility proportion of the crude drugs (the ratio of *Bombyx batryticatus* vs *Periostracum cicada* vs *Curcuma longa* vs raw rhubarb was 2 : 1 : 3 : 4) and reconstituted with distilled water at a concentration of 1 g/ml for the crude drug. The experimental mice were treated with SJP at a concentration of 5 g/kg.BW, which is 10-fold dose of an adult according to the equivalent dose ratio by surface area.

### 2.5. Animals and Treatment

Male C57BL/6 mice weighting 18–20 g were provided by Charles River (Beijing, China). All mice were kept under controlled temperature (22-23°C) and on a 12 h light/12 h dark cycle and followed by free access to a HFD to induce NAFLD or a control diet. All animal feed were provided by Trophic Animal Feed High-tech Co., Ltd (Jiangsu, China). The specific information about the animal feed can be found at http://trophic.biomart.cn. In brief, the HFD we used was TP23300 with 60% of calories derived from fat, and the control diet was TP23302 with 16.7% of calories derived from fat. Animals were randomly allocated to the control group (AC, control diet, *n* = 7), NAFLD group (AT, high-fat diet, *n* = 7), and SJP treatment group (AS, high-fat diet plus SJP treatment, *n* = 7) by random number table. Special feeding lasted for 12 weeks, and the mice in the SJP group were gavaged with SJP once a day from the beginning of the seventh week, while mice in another two groups received equal volume of normal saline instead. We recorded the body weight every 3 weeks, and the fecal samples were collected under a sterile environment for gut microbiota analysis after 12-week feeding. All mice were sacrificed by breaking the neck. The blood and tissue samples were obtained for biochemical test, histopathological analysis, PCR, and Western blotting.

### 2.6. Serum Biochemical Test and Inflammatory Cytokine Analysis

All mice were fasted for 12 h before getting blood samples from the inner canthus vein. The blood samples were then centrifuged at 2500 rpm for 5 min, and the supernatants were collected for biochemical test and inflammatory cytokines analysis. Serum transaminase and lipid were tested by an automatic biochemical analyzer (HITACHI, 7170A, Japan), and serum IL-6 and IL-10 were analyzed by enzyme-linked immunosorbent assay (ELISA) with commercially available materials (eBio, Wuhan, China). According to the manufacturer's protocol, absorbance was measured at 450 nm with High-Throughput Universal Microplate Assay. The sample values were then read off the standard curve, and the relative concentrations were calculated.

### 2.7. Real-Time Quantitative Polymerase Chain Reaction (qRT-PCR)

Expressions of sterol regulatory element-binding protein 1c (SREBP1c) and peroxisome proliferator-activated receptor *γ* (PPAR*γ*) were quantified by qRT-PCR analysis. In brief, total RNA was extracted from fresh liver using TRIzol (15596018, Invitrogen) and reverse-transcribed into cDNA using an iScriptcDNA synthesis kit (Bio-Rad, USA). Real-time qPCR was performed using a Bio-Rad nucleic acid electrophoresis apparatus (Bio-Rad CFX96) and Bio-Rad gel imaging system (GelDoc XR). The primers for the target genes were synthesized by Bohao Biotechnology Co., Ltd. (Shanghai, China). The primer sequences used in this study were as follows: SREBP1c (forward: 5′-CTTTGGCCTCGCTTTTCGG-3′; reverse: 5′-TGGGTCCAATTAGAGCCATCTC-3′) and PPAR*γ* (forward: 5′-CTCCAAGAATACCAAAGTGCGA-3′; reverse: 5′- GCCTGATGCTTTATCCCCACA-3′). GAPDH was used as the internal control. Relative fold changes in gene expression were determined by the 2^−ΔΔ*Ct*^ method.

### 2.8. Oil Red O Staining

Fresh liver samples were immediately embedded in tissue freezing medium (SAKURA Tissue-Tek O.C.T. Compound, Order Number 4583, USA) and absolutely frozen to a solid by a frozen slicer. Then, the frozen liver samples were sectioned into 5 *μ*m slices and fixed by 4% paraformaldehyde for 10 minutes. Afterwards, the frozen liver slices were washed with phosphate buffer saline (PBS) twice and then stained with a freshly prepared working solution of Oil Red O (the stock solution was prepared with 0.5 g Oil red O dissolved in 100 ml isopropanol, and then, the working solution was prepared by adding distilled water to the stock solution at a proportion of 3 : 2) for 1 h at room temperature followed by being counterstained with hematoxylin before microscopic observation (Olympus BX63, Tokyo, Japan). And the images were captured using Cytation 5 cell Imaging Multimode reader (BioTek Instrument, USA).

### 2.9. 16s rDNA Bioinformatics Analysis

Genomic DNA was extracted from fecal samples using the MoBio Power Fecal DNA isolation kit (Qiagen, Germantown, MD) and then was detected by 1% agarose gel electrophoresis. Specific primers with barcode were synthesized according to the specified sequencing region of 16s rRNA gene for PCR amplification. PCR was performed using TransStart Fastpfu DNA Polymerase (TransGen AP221-02), and the PCR products were detected using QuantiFluor™-ST blue fluorescence quantitative system (Promega). All samples were sequenced in triplicate on the Illumina MiSeq platform (Illumina, San Diego, CA). Sequencing data were processed by using Quantitative Insights Into Microbial Ecology (QIIME, v.1.9.1, http://qiime.org/scripts/assign_taxonomy.html). PE reads obtained from MiSeq sequencing were first spliced according to the overlap relationship. At the same time, the sequence quality is controlled and filtered, and then, OTU clustering analysis and species taxonomy analysis were carried out after differentiating samples. We joined reads using a minimum overlap of 10 bp and a maximum percent difference within the overlap of 20%. Operational taxonomic units (OTUs) were assigned by clustering sequence reads at 97% similarity via Usearch platform (version 7.0, http://drive5.com/usearch/), and the chimeras were removed during the clustering process to obtain the OTU representative sequences. Then, OTUs were aligned against the Silva database (Release128, http://www.arb-silva.de). Taxonomic assignment was completed using the RDP classifier (version 2.2, http://sourceforge.net/projects/rdpclassifier/) with a confidence threshold of 0.7. Shannon rarefaction curve was made to make sure the sequencing depth was sufficient to capture biodiversity in the tested samples. Then, within-sample diversity (*α*-diversity) was evaluated by Shannon, Simpson, Chao, and Ace indexes. Between-sample diversity (*β*-diversity) was measured by hierarchical clustering of distance matrix. Ordination of *β*-diversity metrics was then visually displayed by principal coordinates analyses (PCoA) and partial least squares discriminant analysis (PLS-DA). Differences in microbial community abundance among three groups of samples at the OTUs and genus levels were examined by the Kruskal–Wallis H test, and *p* < 0.05 was considered statistically significant.

### 2.10. Immune Blotting of SREBP1c, LXR, FASN, and PPAR*γ*

Expression of SREBP1c, liver X receptor (LXR), fatty acid synthase (FASN), and PPAR*γ* were determined by Western blot analysis. In brief, fresh tissue samples pretreated with appropriate amount of RIPA buffer containing PMSF underwent a ultrasonication and then were centrifuged at 13000 rpm for 10 minutes. The supernatants were collected for total protein quantification. The protein concentrations were then determined using the Bio-Rad protein assay kit (Bio-Rad Laboratories) according to the manufacturer's instructions. The lysates were then separated on an 8% SDS/PAGE. Following electrophoretic transfer on to nitrocellulose membranes and blocking with 5% milk solution, blots were incubated overnight at 4°C with primary rabbit polyclonal/monoclonal antibodies against SREBP1c (1 : 1000, #9874, Cell Signaling Technology), LXR (1 : 1000, #ab176323, Abcam), FASN (1 : 1000, #3189, Cell Signaling Technology), and PPAR*γ* (1 : 1000, #2430, Cell Signaling Technology) and with a secondary antibody conjugated with horseradish peroxidase (Bio-Rad Laboratories) for 3 h at room temperature. Membranes were processed for protein detection using SuperSignal substrate (Pierce), and anti-GAPDH (ABD Serotec) was used as the loading control.

### 2.11. Histopathological Analysis

Fresh tissue samples were fixed in 10% neutral formalin and embedded in paraffin and then sectioned into 5 *μ*m slices and followed with hematoxylin and eosin (H and E) staining. All the histopathology specimens were reviewed and scored in a blinded fashion by two independent pathologists using a scoring system for the extent and severity of tissue injury (points 0–4, steatosis, inflammatory infiltration, necrosis, and fibrogenesis) as previously described [26]. The total histopathology score is the mean of the combined scores for each parameter from both investigators.

## 3. Statistical Analysis

All data were expressed as mean ± SD. Statistical analysis was performed with PEMS3.1 statistical program for Windows. One-way ANOVA was used to analyze group differences in the study. Differences with a *p* < 0.05 were considered to be statistically significant.

## 4. Results

### 4.1. SJP Protected against HFD-Induced Liver Injury

**Figure 1:**
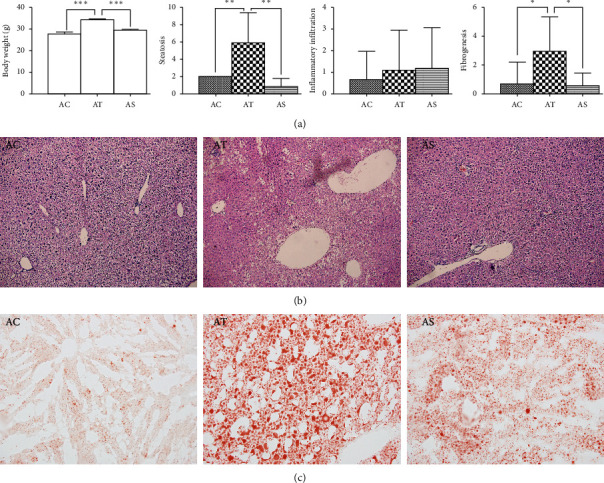
Comparation of body weight and histopathological injuries among groups. (a) Statistical analysis of body weight and pathological score. AC: control group; AT: NAFLD group; AS: SJP treatment group. ^*∗*^*p* < 0.05, ^∗∗^*p* < 0.01, and ^∗∗∗^*p* < 0.001. (b) Pathological images of liver among diverse groups. Hematoxylin-eosin counterstain. Histological images are presented with original magnifcation 200x. Liver in HFG exhibited enlarged hepatocytes and extensive vacuolization which indicated liver steatosis in the AT group, and these changes were ameliorated in the AS group. (c) Oil red O staining of frozen liver sections. The frozen liver sections showed significant lipid droplets accumulation in the AT group, and the lipid droplets accumulation decreased dramatically in the AS group.

**Figure 2:**
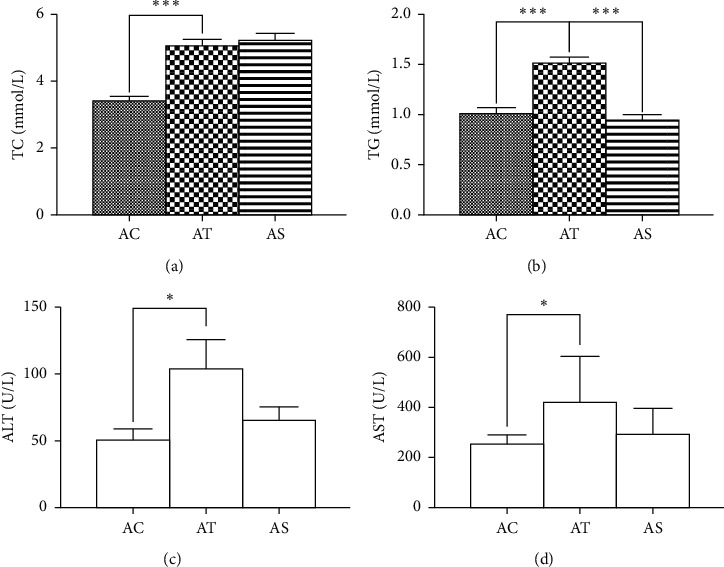
Serum levels of TG, TC, AST, and ALT. AC: control group; AT: NAFLD group; AS: SJP treatment group. ^*∗*^*p* < 0.05 and ^∗∗∗^*p* < 0.001.

HFD feeding induced a significant weight gain at the end of the intervention period and treatment with SJP effectively prevented body weight increase. Histopathological injuries induced by HFD feeding were evaluated by steatosis, inflammatory infiltration, necrosis, and compensatory fibrogenesis. Although there was no difference in inflammatory cellular infiltration among the three experimental groups, steatosis and compensatory fibrogenesis were much severe in HFD feeding mice. Plus, oil red O staining showed that there were more lipid droplets accumulation in mice liver samples from the AT group, while SJP can dramatically reduce liver lipid droplets accumulation and mitigate steatosis and fibrogenesis (Figure 1). Also, we observed a significant increase in serum levels of triglyceride (TG), total cholesterol (TC), alanine aminotransferase (ALT), and aspartate aminotransferase (AST) in HFD feeding mice (Figure 2), but there was no difference observed in serum levels of IL-6 and IL-10 between AC and AT groups (data not shown). SJP treatment decreased serum levels of TG, AST, and ALT, but only the TG level was statistically different between AT and AS groups.

### 4.2. Effect of SJP on Expression of LXR, SREBP1-c, FASN, and PPAR*γ*

**Figure 3:**
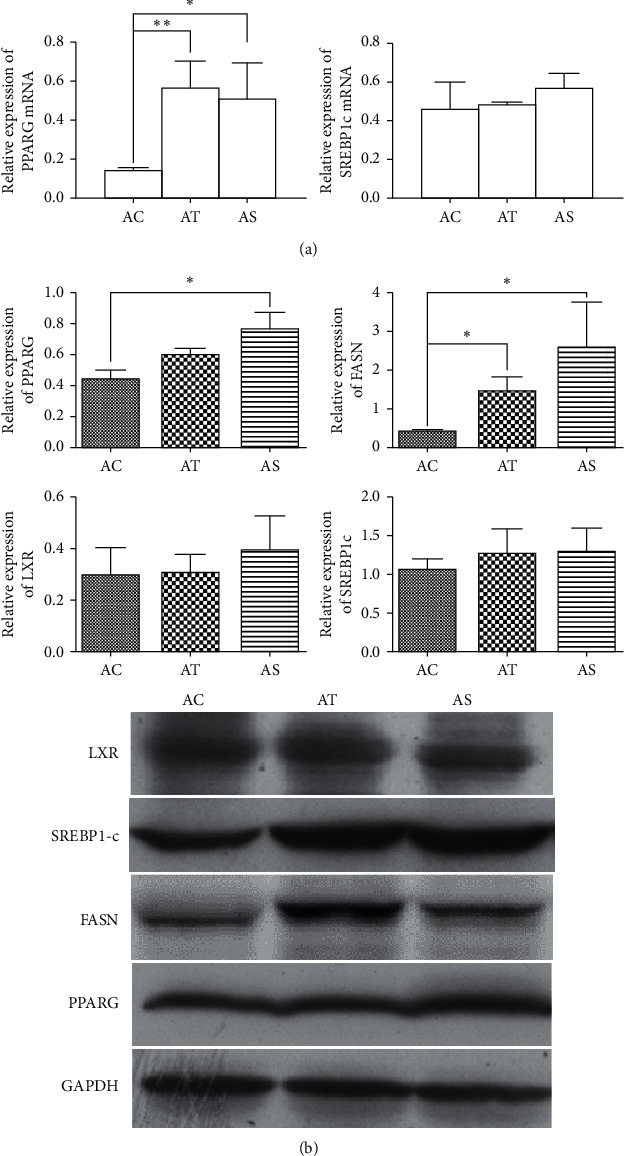
Expression of LXR, SREBP1-c, FASN, and PPAR*γ*. (a) Expression of SREBP1-c mRNA and PPAR*γ* mRNA quantified by PCR. (b) Western blot analysis of LXR, SREBP1-c, FASN, and PPAR*γ* in liver samples among diverse groups. ^*∗*^*p* < 0.05 and ^∗∗^*p* < 0.01.

SREBP1c and PPAR*γ* are both important regulators of lipid metabolism and have significant effect on regulating lipogenetic gene expression. Compared with the control mice, we found a significant increase in PPAR*γ* mRNA expression in HFD feeding mice with/without SJP treatment, but there was no statistical difference in SREBP1c mRNA expression among diverse groups. Likewise, there was no difference in the expression of LXR, an upstream regulator of SREBP1c, but we found a significant increase in expression of downstream FASN in liver tissues of HFD feeding mice. Interestingly, although there was a significant increase in PPAR*γ* mRNA expression in HFD feeding mice, the protein level of PPAR*γ* did not increase in those without SJP treatment (Figure 3).

### 4.3. SJP Changed the Altered Gut Microbiota Diversity Induced by NAFLD

#### 4.3.1. Analysis of the Gut Microbiota Diversity

**Figure 4:**
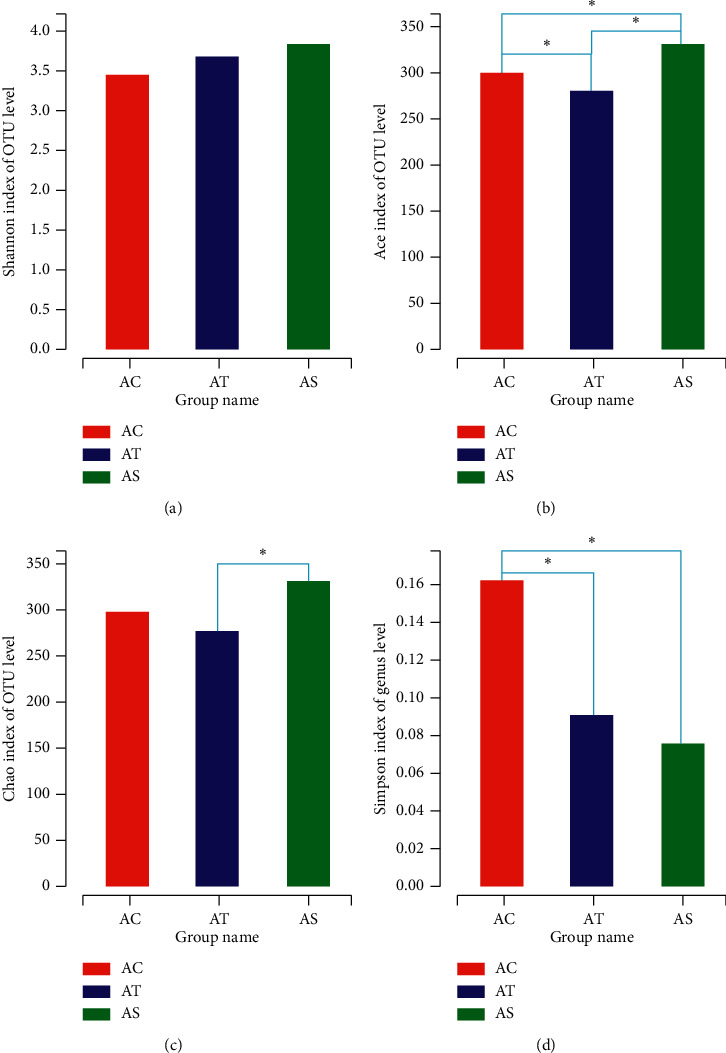
*α*-Diversity analysis of gut microbiota among the three experimental groups. Shannon and Simpson indexes indicate the community diversity (a, d), and Chao and Ace indexes indicate the community richness (b, c). ^*∗*^*p* < 0.05.

**Figure 5:**
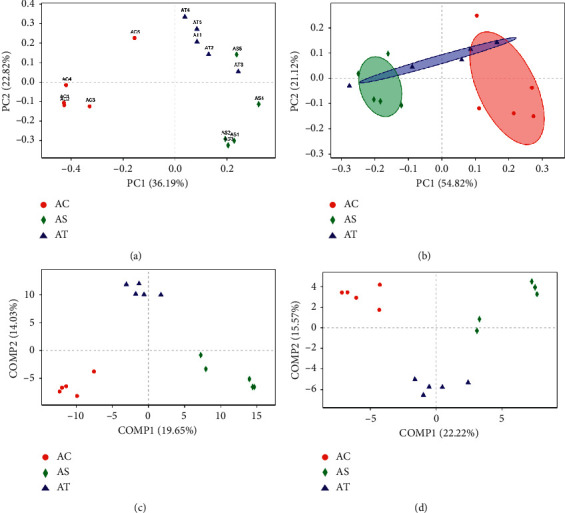
Principal coordinates analysis (PCoA) and partial least squares discriminant analysis (PLS-DA) for unweighted uniFrac distance metric in gut microbiota among diverse groups at both OTU and genus level. (a) PCoA on OTU level; (b) PCoA on genus level; (c) PLS-DA on OTU level; (d) PLS-DA on genus level.

Gut microbiota diversity was evaluated in the setting of smooth Shannon rarefaction curves, demonstrating the sequencing depth was sufficient to capture biodiversity in the tested samples (data not shown here). The *α*-diversity was reflected by Shannon and/or Simpson index, indicating the community diversity and Chao and/or Ace index, indicating the community richness. The NAFLD mice had an obvious lower community richness than the control, while SJP can effectively alter this situation (Figures 4(b) and 4(c)). Although the Shannon index showed no difference among the three groups in community diversity in the OTU level (Figure 1(a)), there was a significant decrease in community diversity at genus level in NAFLD mice whether with or without SJP treatment (Figure 4(d)). PCoA and PLS-DA of the gut microbiota diversity revealed apparent clustering among the three experimental groups at both OTU and genus level, indicating that the gut micorobiota diversity changed in NAFLD mice and SJP was effective in modulating gut micorobiota diversity (Figure 5).

#### 4.3.2. Analysis of the Gut Microbiota Composition

**Figure 6:**
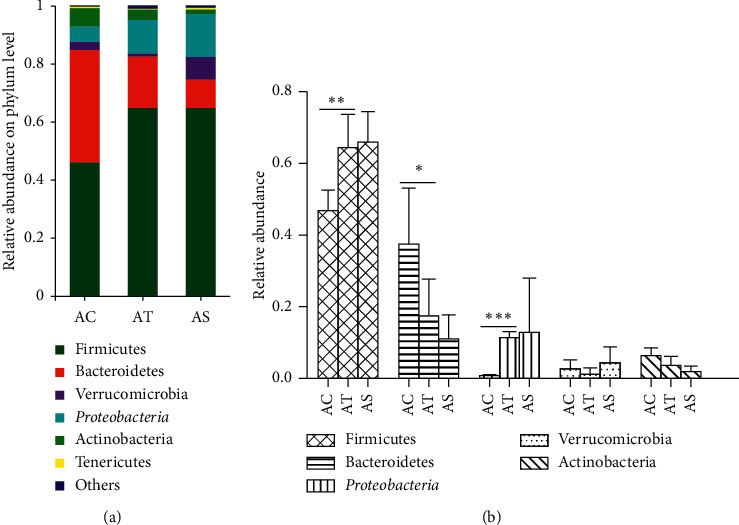
Relative abundance of intestinal microbiota at phylum level. Species with less than 1% abundance was classified into the “others” category. Differences in the microbial community abundance among the three groups were analyzed by one-way ANOVA. ^*∗*^*p* < 0.05, ^∗∗^*p* < 0.01, and ^∗∗∗^*p* < 0.001.

At the phylum level, the majority of the gut microbiota consisted of species from *Firmicutes*, *Bacteroidetes*, *Verrucomicrobia*, *Proteobacteria*, *Actinobacteria*, and *Tenericutes* (Figure 6(a)). The microbial composition altered in NAFLD mice with significant increased abundance of *Firmicutes* and *Proteobacteria* and simultaneously decreased abundance of *Bacteroidetes*. The abundance of *Verrucomicrobia* and *Actinobacteria* decreased in NAFLD mice, and SJP showed potential to increase the abundance of *Verrucomicrobia*, but there was no statistical difference (Figure 6(b)). Additionally, we observed a decreased ratio of *Firmicutes*/*Bacteroidetes* in AT and AS groups, which were the top 2 major species in each group (the ratio was 0.28 in the AT group, 0.16 in the AS group, and 0.84 in the AC group). And the ratio might be related to obesity and related disease according to the emerging evidence.

**Figure 7:**
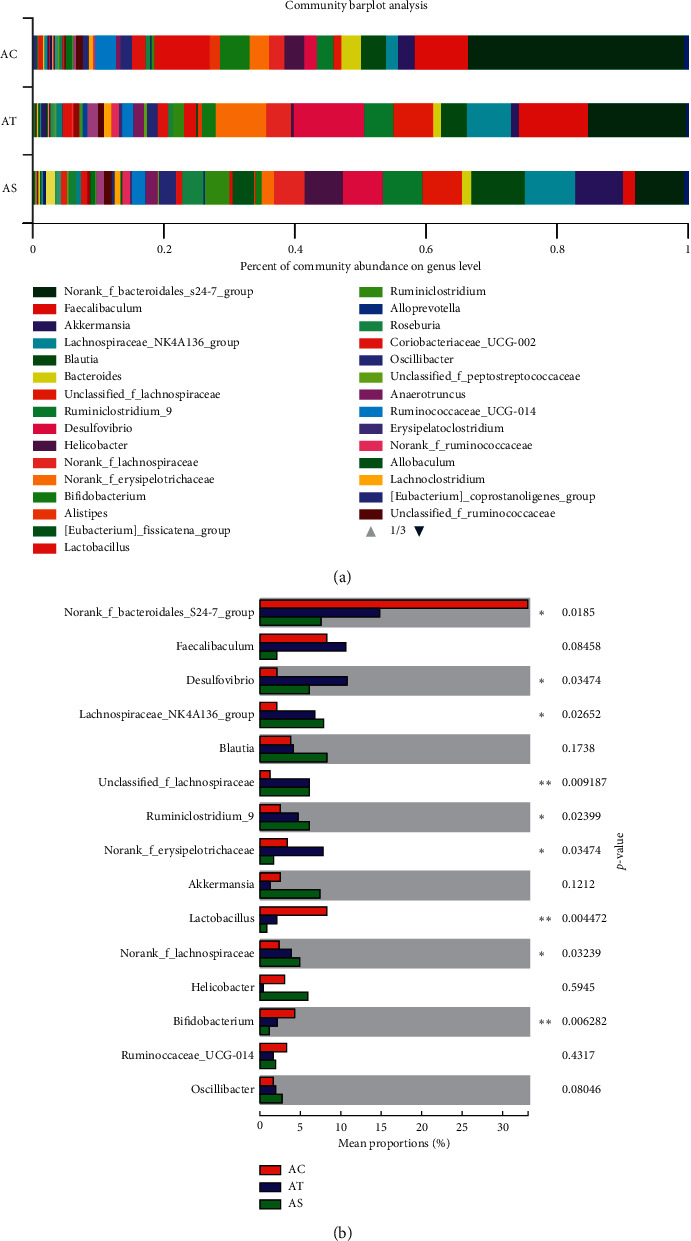
Relative abundance of intestinal microbiota at genus level. (a) Top 40 high abundant species at the genus level. (b) Top 15 high abundant species at the genus level. Differences in the microbial community abundance among the three groups were analyzed by the Kruskal–Wallis H test. ^*∗*^*p* < 0.05; ^∗∗^*p* < 0.01.

**Figure 8:**
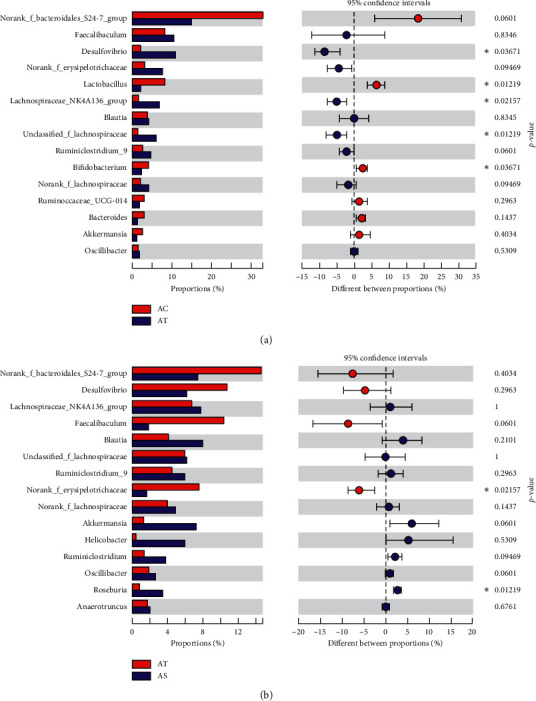
Top 15 high abundant species at the genus level. Differences in the microbial community abundance between groups (AC vs AT and AT vs AS) were analyzed by Wilcoxon rank-sum test. ^*∗*^*p* < 0.05.

The species community composition of the three experimental groups at the genus level appeared to be different too (Figure 7(a)). In our study, we selected the top 15 high abundant species to analyze their express in each group. We found species like *Faecalibaculum*, *Blautia*, norank-f-*Bacteroidales*-S24-7-group, *Desulfovibrio*, *achnospiraceae*-NK4A136-group, *Lactobacillus*, unclassified-f-*Lachnospiraceae*, *Ruminiclostridium*-9, norank-f-*Erysipelotrichaceae*, norank-f-*Lachnospiraceae*, and *Bifidobacterium* significantly contributed to the between group differences among the three groups (Figure 7(b)). To further identify the species that discriminate NALFD and the control or SJP-treated mice, differences in the microbial community abundance between groups (AC vs AT and AT vs AS) were analyzed by the Wilcoxon rank-sum test. Compared with the AC group, the abundance of *Desulfovibrio*, unclassified-f-*Lachnospiraceae*, and *Lachnospiraceae*-NK4A136-group was significantly increased in the AT group (10.69%, 5.99%, and 6.75% abundance in the AT group and 2.08%, 1.17%, and 1.81% abundance in the AC group, *p* < 0.05), while the abundance of *Lactobacillus* and *Bifidobacterium* was significant decreased (1.92% and 2.10% abundance in the AT group and 8.37% and 4.35% abundance in the AC group, *p* < 0.05). The abundance of *Desulfovibrio* decreased after being treated with SJP, but there was no significant statistic difference (the corresponding abundance of *Desulfovibrio* was 6.03%). Additionally, we found increased abundance of *faecalibaculum* and norank-f-*Erysipelotrichaceae* and decreased abundance of *Helicobacter* and *Akkermansia* in the AT group, while these species appeared to be changed in the opposite direction in the AS group. To our surprise, we found a significant increased abundance of *Roseburia* in the AS group which was not included in the top 15 species at the genus level (Figure 8).

#### 4.3.3. Function Prediction of the Gut Microbiota

**Figure 9:**
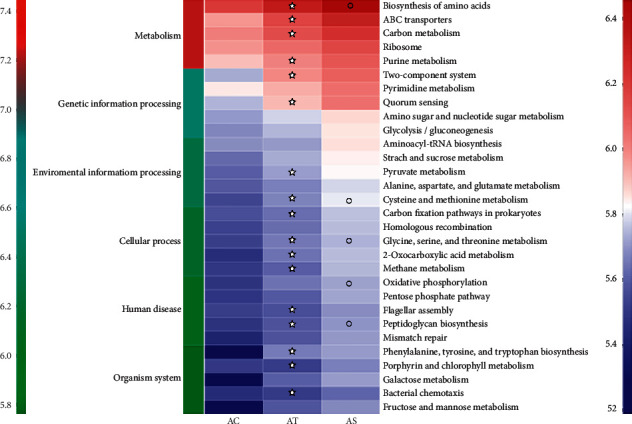
Function prediction of the gut microbiota. Top 30 high abundant species were included for function prediction using PICRUSt2. The *x*-axis indicates the sample groups, the *y*-axis indicates different pathways (left green-blue-red gradient bar represents pathway level 1, and right blue-white-red gradient bar represents pathway level 3). ✮, compared to the AC group, *p* < 0.05; ○, compared to the AT group, *p* < 0.05.

The OTU abundance was further analyzed by PICRUSt2 for function prediction. The top 30 high abundant species were involved in 6 metabolic pathways, and species with the highest abundance was enriched in metabolism pathway, including biosynthesis of amino acids, ABC transporters, carbon metabolism, ribosome, and purine metabolism. According to our results, the changed gut microbiota in the AT group were likely to affect a series of pathways, and SJP seemed to have a greater impact on these pathways, especially on biosynthesis of amino acid, cysteine and methionine metabolism, glycine, serine, and threonine metabolism, oxidative phosphorylation, and peptidoglycan biosynthesis (Figure 9).

## 5. Discussion

In the present study, HFD feeding induced a series of clinical features of NAFLD, including obesity, hyperlipidemia, liver function injury, and hepatic steatosis. SJP treatment protected against the increase in body weight and serum TG level, showed potential to improve liver function, and finally improved the pathological state of NAFLD mice. Additionally, increased expression of PPAR*γ* mRNA was observed in HFD feeding mice, but a steady elevation of PPAR*γ* protein level was only found in SJP-treated mice. Increased expression of FASN was also found in HFD feeding mice, while there seemed to be no difference in the expression of SREBP1-c and LXR.

Lipid synthesis in the liver is a strictly regulated process that is important for the formation of very low-density lipoproteins and the delivery of energy to other tissues. Excessive supply of fatty acid induces the formation of triglyceride, and then, lipid droplets accumulate in hepatocytes when triglyceride cannot be fully exported into the blood, which consequently leads to the development of NAFLD [27]. Transcription of lipid synthesis genes is regulated by a series of transcription factors such as steroid response element-binding proteins (SREBPs), liver X receptors (LXRs), and peroxisome proliferator-activated receptors (PPARs). SREBP1c is thought to be the most important regulator of lipogenic gene expression among the three subtypes of SREBPs [27]. Current studies reveal that the expression of SREBP1c is generally elevated in obese mice with insulin resistance and hepatic steatosis. LXR is another regulator of lipogeneic gene expression that activates lipogenic gene directly or indirectly inducing SREBP1c activation [28, 29]. Likewise, PPAR*γ* also plays an important role in regulating lipid metabolism, especially in HFD feeding induced lipogeneic gene expression and hepatic lipid biosynthesis [30]. However, FANS is a multipeptide enzyme that is mainly responsible for the synthesis of palmitate, a long-chain saturated fatty acid. In the present study, HFD feeding induced increased expression of FASN, serum lipid accumulation, and hepatic lipid deposition in mice, but the expression of upstream transcription factors LXR and SREBP1c was not changed. Except for obvious liver function injury, it is in accordance with our previous rat models of NAFLD with a higher expression of FASN and unchanged expression of SREBP1-c, and the difference might be due to different experimental species. SJP was demonstrated to be efficient in anti-inflammation, lowering body weight and blood lipid and mitigating insulin resistance. Herein, SJP showed great potential to protect against HFD-induced metabolic changes as expected. Additionally, increased expression of PPAR*γ* mRNA was observed in HFD feeding mice whether or not treated with SJP, but a steady elevation of PPAR*γ* protein was only found in SJP-treated mice. This result will partly interpret the effect of SJP on improving insulin resistance as PPAR*γ* is a transcription factor closely associated with insulin resistance.

The gut microbiota consist of trillions of microorganisms that affect body's physiological metabolism by interacting with different environmental conditions, such as diet, alcohol intake, antibiotic abuse, and hereditary factors [31] And these perturbations can result in a state of dysbiosis of the ecosystem [32]. Accumulated evidence has demonstrated that dietary fats intake might affect gut microbiota composition. HFD feeding was reported to induce changes in the intestinal microbiota composition in animal models, and the changed species was found to be closely related to obesity-associated metabolic parameters [33, 34]. According to the present studies, *Bacteroidetes* and *Firmicutes* were the most abundant species in the host microbiota and they played important roles in host metabolism. The increased ratio of *Firmicutes/Bacteroidetes* was related to obesity due to a higher capacity to harvest energy from the diet, thereby supplying more substrates for lipogenic pathways activation [33, 35]. In our study, we found that the proportion of *Firmicutes* and *Proteobacteria* was significantly increased in HFD feeding mice, while the proportion of *Bacteroidetes* was significantly decreased. We also observed a decrease in gut microbiota diversity in HFD feeding mice as the Ace index and Simpson index were much lower than the control mice. These changes in microbiota composition and diversity were in accordance with most of the current evidence [35, 36]. Of note, coadministration of SJP did not reverse the increased ratio of *Firmicutes/Bacteroidetes*, but it did protect against hepatic steatosis, hyperlipidemia, and obesity in HFD feeding mice. This might be partly attributed to the increased community richness.

At the genus level, we found that the majority of the species changed among the three experimental groups were involved in short-chain fatty acids (SCFAs) producing family, such as unclassified-f-L*achnospiraceae*, *Lachnospiraceae*-NK4A136-group, *Faecalibacterium*, *Akkermansia*, *Bifidobacterium*, *Lactobacillus,* and *Roseburia*. As the end product of bacterial fermentation of soluble dietary fiber and indigestible carbohydrates, SCFAs might be able to regulate a series of process that alters energy harvest and metabolism, such as regulating appetite, promoting energy consumption, stimulating insulin sensitivity, and activating adenosine monophosphate-activated protein kinases (AMPK) in the liver and skeletal muscle, and thereby affecting the development of NAFLD [37]. In the present study, we found a HFD-induced decrease in the abundance of *Bifidobacterium* and *Lactobacillus* whose major function was participating in the metabolic process including anaerobic conversion of polymeric sugars into short-chain fatty acids (SCFAs) [38–40]. We also found an HFD-induced increase in the abundance of unclassified-f-*Lachnospiraceae*, *Lachnospiraceae*-NK4A136-group, and *Desulfovibrio*. Increased abundance of *Desulfovibrio* was considered to be associated with intestinal disorders [39]. And unclassified-f-*Lachnospiraceae* and *Lachnospiraceae*-NK4A136-group are both members of *Lachnospiraceae* family which are the main producers of SCFAs. Their changes in the present study aroused our attention as they were supposed to decrease according to the current evidence. Interestingly, their abundance was even higher in SJP-treated mice. Thus, their role in lipid metabolism may need further investigation.

The mechanism of SJP protecting against HFD-induced obesity and hepatic steatosis might be attributed to the regulation of SCFA-producing process as we found a remarkable decrease in the abundance of norank-f-*Erysipelotrichaceae* and increase in the abundance of *Roseburia* in SJP-treated mice. The above two species are both members of *Firmicutes* phylum and involved in the SCFA producing process. The former belongs to *Erysipelotrichaceae* family, while the latter belongs to *Lachnospiraceae* family. Current knowledge on the role of *Erysipelotrichaceae* and *Lachnospiraceae* in human disease mainly comes from studies focused on metabolic disorders and nutrition [41]. Generally, increased abundance of the members of *Erysipelotrichaceae* and *Lachnospiraceae* family was observed in obese individuals [42]. Also, high-fat diet induced increase in the abundance of *Erysipelotrichaceae* and *Lachnospiraceae* family [43]. Furthermore, a positive correlation with the abundance of *Erysipelotrichaceae* and liver fat was found in individuals on high-fat and choline deficiency diet [44]. An obvious decrease in the abundance of *Erysipelotrichaceae* was observed accompanied with lose in body weight, decline in blood lipids, and reduction in liver injury after treating with cholesterol lowing drugs, antibiotics, and some herbal extracts [45, 46]. Dietary intake also influences the abundance of *Roseburia* [47]. Current evidence found that the abundance of *Roseburia* was negatively correlated with high fat induced body weight increase, fat mass development, and hepatic lipid deposition [48]. However, Alejandra and colleagues found that *Roseburia* was significantly more abundant in obese Mexican women with or without metabolic disorder, while the abundance of *Erysipelotrichaceae* family was significantly decreased [49]. These different findings will promote further studies to explore the unknown effect of gut microbiota.

Function prediction of the top 30 species with highest abundance by PICRUSt2 suggested the enrichment of bacteria in the metabolism pathway. HFD feeding induced enhancement of the majority of the enriched biological pathways, while SJP appeared to have a greater impact on these pathways, especially on biosynthesis of amino acid, cysteine and methionine metabolism, glycine, serine, and threonine metabolism, oxidative phosphorylation, and peptidoglycan biosynthesis. Protein, lipid, and glucose metabolism were mainly involved in the above pathways [50]. In other words, SJP might have a deeper impact on the metabolic process of protein, lipid, and glucose. As the biological functions of the floras were complex, the mechanism of how the changed gut microbiota affect metabolism of nutrients and the development of NAFLD still need further investigation.

Herein, we established a mice model of NAFLD without obvious liver inflammation and fibrosis. The relevant parameters we detected and the effect of SJP we observed were limited to simple steatosis mice models. Further studies on more severe state of NAFLD will promote our understanding on the effect of SJP on gut microbiota.

In conclusion, SJP protected against HFD-induced NAFLD and this effect might be partly attributed to the regulation of gut microbiota.

## Data Availability

The raw data can only be obtained by emailing the authors because some unpublished results are included in it.
